# Long-Term NMDAR Antagonism Correlates Weight Loss With Less Eating

**DOI:** 10.3389/fpsyt.2019.00015

**Published:** 2019-02-08

**Authors:** Shi-Ning Deng, Yu-Hua Yan, Tai-Lin Zhu, Bing-Ke Ma, Hui-Ran Fan, Yan-Mei Liu, Wei-Guang Li, Fei Li

**Affiliations:** ^1^Developmental and Behavioral Pediatric Department and Child Primary Care Department, Ministry of Education-Shanghai Key Laboratory of Children's Environmental Health, Shanghai Institute for Pediatric Research, Xinhua Hospital Affiliated Shanghai Jiao Tong University School of Medicine, Shanghai, China; ^2^Key Laboratory of Brain Functional Genomics, Ministry of Education, East China Normal University, Shanghai, China; ^3^Collaborative Innovation Center for Brain Science, Department of Anatomy and Physiology, Shanghai Jiao Tong University School of Medicine, Shanghai, China

**Keywords:** memantine, obesity, weight loss, high fat food, NMDA (N-methy-D-aspartate receptor)

## Abstract

Memantine hydrochloride is an uncompetitive N-methyl-D-aspartate (NMDA) antagonist for treatment of moderate-to-severe Alzheimer's disease. Several studies have shown that memantine can significantly correct the binge-like eating behavior in human and animal models. People with overeating behavior are more likely to be obese. Therefore, we suppose that memantine would be a good candidate for the treatment of obesity. In this study, memantine was shown to increase weight loss in obese mice induced by high fat diet. Memantine was shown to decrease food intake without inducing abdominal discomfort and anxiety, suggesting that this compound would be a good candidate drug for obesity control.

## Introduction

According to WHO 2018, worldwide obesity has nearly tripled since 1975 (http://www.who.int/mediacentre/factsheets/fs311/en/). Orlistat was the only available weight-loss medicine since 1999. Recently, four new anti-obesity drugs (lorcaserin, phentermine/topiramate, altrexone/bupropion and liraglutide 3.0 mg) for long-term use were approved in the USA. The new drugs were clinically effective, but their high price and risk of adverse effects shouldn't be ignored (1). Thus, new candidate drugs are still urgently needed.

Memantine hydrochloride, an uncompetitive N-methyl-D-aspartate receptor (NMDAR) antagonist, was approved in Europe (in 2002, marketed under the product names Ebixa and Axura) and the US (in 2003, marketed as Namenda) for moderate-to-severe Alzheimer's disease (AD) (2). Memantine is not only used for neurodegenerative diseases, but also for some neuropsychiatric syndromes, like binge eating disorder. Several studies have shown that memantine can significantly correct the binge-like eating behavior in human and animal models (3–6). As we know, our eating behavior can decide our whole day caloric intake. Eating behavior plays important role in obesity by modulating hormones such as letpin and ghrelin, which are related to BMI and body fat (7, 8). The imbalance of leptin and ghrelin affects the brain rewards system and promotes overeating (7, 8). People with overeating behavior are more likely to be obese (9–12). Therefore, we suppose that memantine would be a good candidate for the treatment of obesity. Further studies on whether and how memantine increases weight loss are needed.

The present study aimed to investigate the effects of memantine on weight loss. By taking advantage of the obesity mouse model, we firstly explored whether long-term NMDAR antagonism by memantine could systemically increase weight loss, and then tried to explain potential mechanisms.

## Materials and Methods

### Animals

Male C57BL/6J mice were housed in standard cages (48 cm × 26 cm), with controlled temperature (22°C) and a 12 h light/12 h dark cycle. There were four mice in each cage. All procedures were carried out in accordance with the guidelines for the Care and Use of Laboratory Animals of Shanghai Jiao Tong University School of Medicine and approved by the Institutional Animal Care and Use Committee [Department of Laboratory Animal Science (DLAS), Shanghai Jiao Tong University School of Medicine] (Policy Number DLAS-MP-ANIM.01–05).

### Long-Term NMDAR Antagonism by Memantine on Obesity

**Figure 1:**
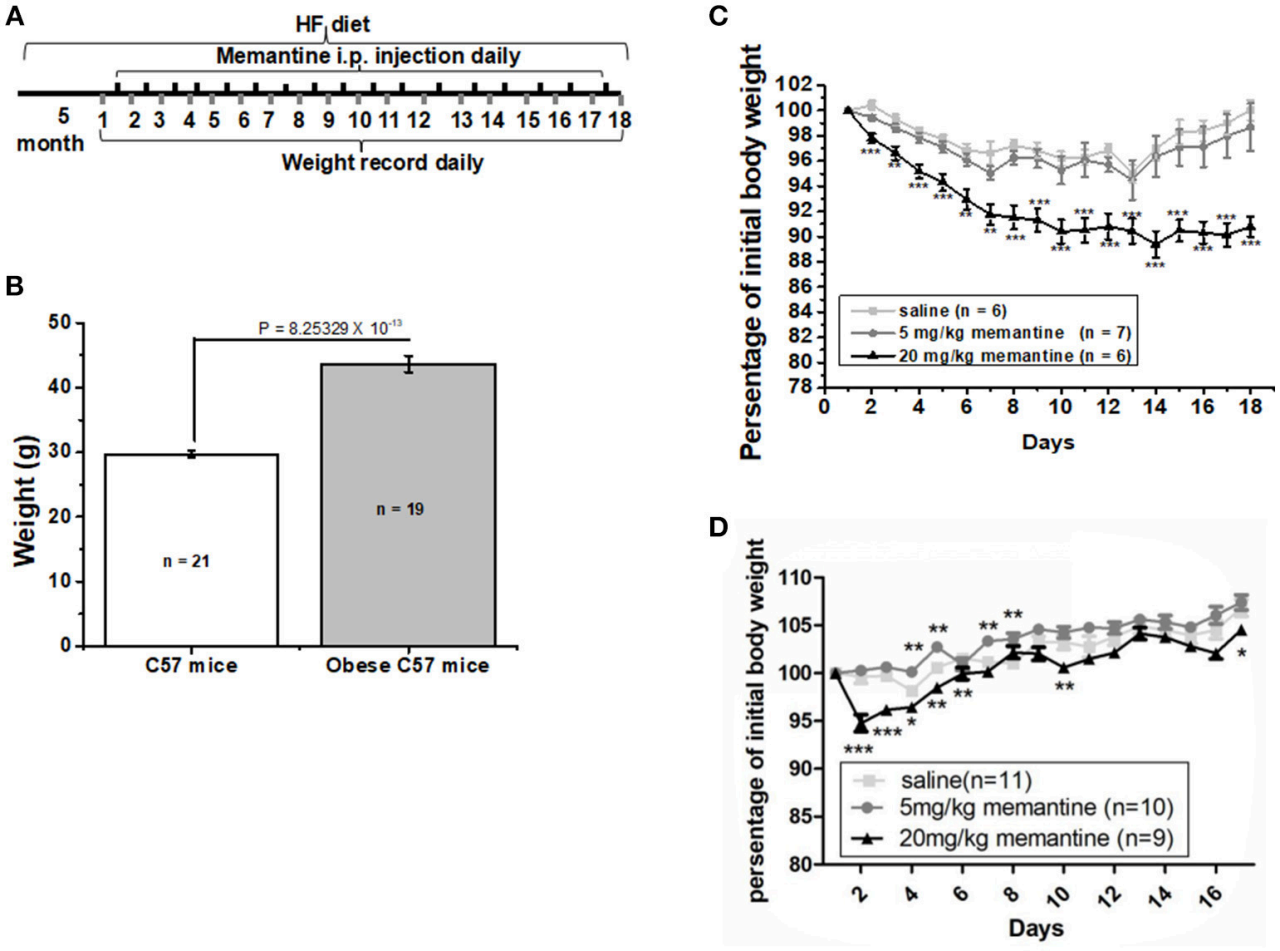
Long-term NMDAR antagonist treatment by memantine decreased the weight of obese mice. **(A)** Schematic representation for memantine injection and weight recording. **(B)** The weight of HF food diet group mice was significantly larger than that of Ctrl food diet group mice (unpaired Student's *t*-test, *p* = 8.25329 × 10^−13^). **(C)** Memantine significantly decreased the percentage of body weight to original body weight during memantine injection days [Day, *F*_(17, 342)_ = 5.577, *P* = 0.000; Group, *F*_(2, 342)_ = 107.155, *P* = 0.000; Day^*^Group, *F*_(51, 342)_ = 1.375, *P* = 0.058, two way ANOVA; from the second day to the end, all *P* < 0.001 except the *P*_3_ day = 0.0028; *P*_6_ day = 0.0023; *P*_7_ day = 0.0027, unpaired Student's *t*-test; **(D)** The percentage of body weight in mice fed with standard control food diet and administered with 20 mg/kg memantine showed a significant decrease during the first memantine injection days but did not differ in late injection days (Day, *F*_(15, 480)_ = 33.133, *P* = 0.000; Group, *F*_(3, 480)_ = 73.964, *P* = 0.000; Day^*^Group, *F*_(51, 342)_ = 1.429, *P* = 0.041, two way ANOVA). ^*^P < 0.05, ^**^P < 0.01, ^***^P < 0.001].

Six-week old C57BL/6J male mice were fed with high fat food (HF group) and standard control food (Ctrl group) for 5 months. The procedure was carried out as illustrated in Figure 1A. The high fat food (HF) contained a total of 45% kcal from fat (D12451, Research Diets, Inc., USA). At the end of the 5 months, the weight of mice in both groups was recorded. Then obese mice were divided into three groups, which were given saline, 5 and 20 mg/kg memantine, respectively. Memantine (Sigma, USA) dissolved in 0.9% saline was injected intraperitoneally for 17 days. During these days, the obese mice were continually fed with HF food. In order to investigate the effects of memantine on C57 mice fed with standard control food, memantine with different doses was also injected intraperitoneally. The body weight during the 17 days was recorded daily.

### Food Intake Test

**Figure 2:**
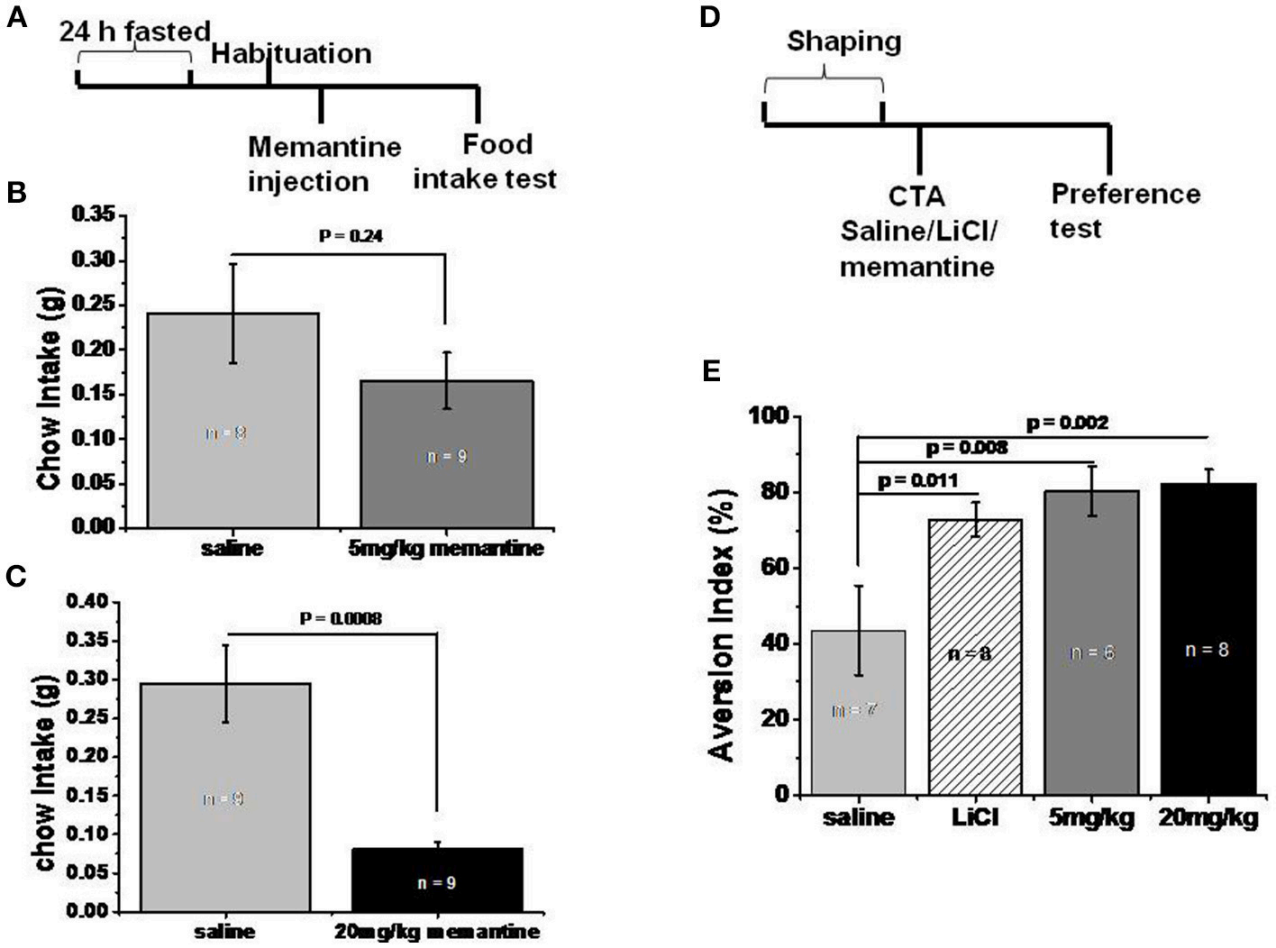
Memantine decreased food intake. **(A)** Schematic representation of standard food intake test protocol by memantine. **(B)** 5 mg/kg memantine group showed similar Ctrl food intake with saline group (unpaired Student's *t*-test, *P* = 0.241). **(C)** 20 mg/kg memantine group significantly reduced the Ctrl food intake compared to saline group (unpaired Student's *t*-test, *P* = 0.0008). **(D)** Schematic representation of CTA behavioral protocol. **(E)** LiCl group and memantine group decreased the intake of sodium saccharin solution [group, *F*_(3, 28)_ = 4.707, *P* = 0.01, one way ANOVA], *post-hoc* analysis revealed LiCl, 5 and 20 mg/kg memantine group increased the aversion index to 72.88% (*P* = 0.011), 74.43% (*P* = 0.008), and 82.29% (*P* = 0.002) respectively, compared to 43.63% of saline group.

Eight-week old C57BL/6J male mice were made to fast for 24 h as previously described (13). The procedure was carried out as illustrated in Figure 2A. Then they were habituated to the test box (mouse cage with new material) for 20 min. After habituation, saline and memantine (5 and 20 mg/kg) were intraperitoneally injected in the home cage. Thirty minute later, standard food was presented in the test box and the food that was consumed during the next 20 min was recorded.

### Conditioned Taste Aversion (CTA) Test

The CTA tests were performed as described previously with some modifications (14). The procedure was carried out as illustrated in Figure 2D. During the 1-week adaptation, 8-week old C57BL/6J male mice drank water once a day from two bottles (from 9:00 to 9:30 a.m.), but had free access to the standard control food. Water intake was recorded for each mouse by weighing both bottles before and after drinking time. Following the adaptation, each mouse was allowed to drink two bottles of 0.5% sodium saccharin solution (0.5% w/v) (Sigma-Aldrich) during the 30 min drinking time. Forty minute after drinking time, mice were given an intraperitoneal injection of saline, LiCl (0.15 M, Sigma-Aldrich), and memantine (5 and 20 mg/kg), respectively. On the day of the test, one bottle of 0.5% sodium saccharin solution and one bottle of water were inserted into each cage simultaneously. Fluid consumption was determined by weighting both bottles before and after drinking time. Aversion index (in %) = water intake (in grams) × 100%/[sodium saccharin intake (in grams) + water intake (in grams)].

### Open Field Test

The test was carried out as previously described (15), in a square plexiglass apparatus (40 × 40 × 40 cm). A digital camera was set above the apparatus. Trace was recorded by the Ethovision video tracking system (Noldus Information Technology, Wageningen, Netherlands). Thirty minute before the test, 9-week old C57BL/6J male mice were intraperitoneally injected with saline and memantine (5 and 20 mg/kg). The mice were then gently placed in the apparatus and were left free to explore for another 60 min. After each trial, the apparatus was cleaned with 75% ethanol. In another 30 min test recorded by Tru Scan system (CoulBourn Instrument, USA), Cholecystokinin (CCK, 30 μg/kg, Tocris Bioscience, USA) and LiCl (150 mg/kg, Sigma-Aldrich, USA) were dissolved in saline and injected intraperitoneally 30 min before test.

### Elevated Plus Maze Test

The protocols were followed as previously described (15). The black plastic elevated plus maze consisted of four 30 cm × 5 cm arms (two open without walls and two enclosed by 15.25 cm high walls). The maze was elevated 40 cm above the floor. Activity was recorded with a digital camera suspended from the ceiling. The test took place during the light phase. On the test day, mice were placed individually in the center of the maze facing the enclosed arms, and recorded for 5 min by the Ethovision tracking system (Noldus Information Technology, Wageningen, Netherlands). The maze was cleaned with 75% alcohol between trials. The time spent in the four arms was analyzed.

### Statistical Analysis

Values were expressed as the mean ±S.E.M. Groups were compared using Student' s *t*-test or ANOVA. *P* < 0.05 was considered to be statistically significant.

## Results

### Memantine Increased the Weight Loss of Obese Mice

After 5 months, the weight of the HF food diet group was significantly larger than that of the Ctrl food diet group (Figure 1B). To investigate the effects of memantine on obesity, memantine was administered intraperitoneally. The percentage of body weight during the 17 memantine injection days compared to the original body weight was analyzed. Compared to the saline group mice, the percentage of body weight in mice fed with HF food diet and administered with 20 mg/kg memantine showed a significant decrease during the memantine injection days (Figure 1C). Meanwhile, the percentage of body weight for 5 mg/kg memantine group mice was similar to that of the saline group mice (Figure 1C). Compared to the saline group mice, the percentage of body weight in mice fed with standard control food diet and administered with 20 mg/kg memantine showed a significant decrease during the early memantine injection days but did not differ in later injection days (Figure 1D). By contrast, the percentage of body weight in mice administered with 5 mg/kg memantine injection was similar with that of saline group mice, while higher in some days (Figure 1D). These data showed the potential of memantine on obesity control.

### Memantine Decreased Food Intake

In order to investigate whether memantine decreased the weight of obese mice by decreasing food intake, mice that fasted for 24 h were intraperitoneally injected with saline and memantine (5 and 20 mg/kg). The 5 mg/kg memantine group showed similar Ctrl food intake to the saline group (Figure 2B). However, the 20 mg/kg memantine group significantly reduced the Ctrl food intake compared to the saline group (Figure 2C). In order to find out whether memantine leads to severe side effect, like abdominal discomfort, CTA model was used. Compared to the saline group, the LiCl, and memantine groups saw a significant decrease in the intake of sodium saccharin solution (Figure 2E).

### Memantine Increased Locomotor Activity Without Severe Side Effect

**Figure 3:**
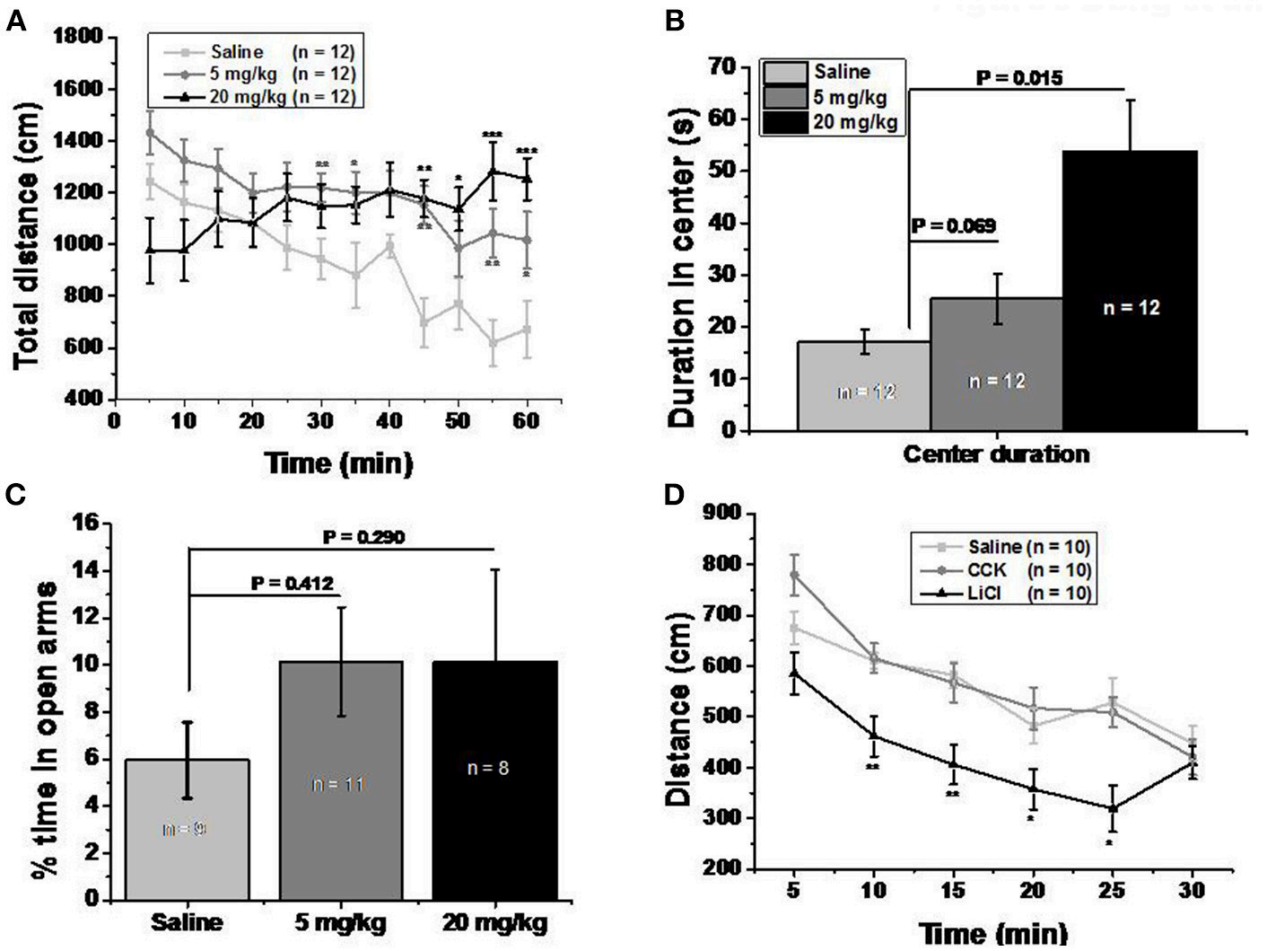
Memantine increased locomotor activity without severe side effect. **(A)** Both 5 and 20 mg/kg memantine group increased the distance during the late period of open field test. [Group, *F*_(2, 360)_ = 26.906, *P* = 0.000; Time, *F*_(11, 360)_ = 2.543, *P* = 0.004; Group^*^Time, *F*_(22, 360)_ = 2.810, *P* = 0.000, two way ANOVA; saline group and 5 mg/kg memantine group, *P*_30_ = 0.009, *P*_35_ = 0.047, *P*_45_ = 0.001, *P*_55_ = 0.004, *P*_60_ = 0.042; saline group and 20 mg/kg memantine group, *P*_55_ = 0.001, *P*_50_ = 0.02, *P*_55_ = 0.0004, *P*_60_ = 0.0008, unpaired Student's *t*-test; ^*^P < 0.05, ^**^P < 0.01, ^***^P < 0.001]. **(B)** Mice injected with memantine spent more time in the center of open field than that of control group mice [group, *F*_(2, 29)_ = 8.652, *P* = 0.001, one way ANOVA; *post-hoc* analysis showed that saline group and 5 mg/kg memantine group, *P* = 0.069; saline group and 20 mg/kg memantine group, *P* = 0.015]. **(C)** Mice injected with memantine spent similar time in open arms with that of saline group mice [group, *F*_(2, 27)_ = 0.64, *p* = 0.536, one way ANOVA; *post-hoc* analysis saline group and 5 mg/kg memantine group, *P* = 0.412; saline group and 20 mg/kg memantine group, *P* = 0.290]. **(D)** LiCl group mice showed significant less distance during the time in open field [group, *F*_(2, 180)_ = 27.270, *P* = 0.000; time, *F*_(5, 180)_ = 20.181, *P* = 0.000; group^*^time, *F*_(10, 180)_ = 0.316; two way ANOVA; saline and LiCl group, *P*_10_ = 0.002, *P*_15_ = 0.002, *P*_20_ = 0.039, *P*_25_ = 0.012, unpaired Student's *t*-test; **(D)**].

Open field test was performed to clarify whether memantine decreased food intake due to abdominal discomfort. Both 5 and 20 mg/kg memantine mice groups showed significantly increased locomotor activity compared to the saline mice group (Figure 3A). Memantine wasn't found to induce anxiety because mice injected with memantine spent more time in the center of the open field than the control group mice (Figure 3B). Besides, in the elevated plus maze test, mice injected with memantine spent similar time in the open arms compared to control group mice (Figure 3C). In order to explore the behavior under satiation and abdominal discomfort condition, CCK and LiCl were used. LiCl group mice covered significant less distance during the time in open field (Figure 3D), while CCK group mice showed a behavior similar to the saline group mice. These results suggest that no abdominal discomfort and no anxiety are induced by memantine.

## Discussion

Our results show that long term NMDAR antagonism by memantine significantly decreased the weight of obese mice. Our results are in accordance with clinical reports. By using an on-off-on design, Schaefer et al. found that memantine discontinuation and re-exposition were followed by a significant weight increase and a substantial weight loss (16). In a therapeutic trial in five obese women, Hermanussen et al. found that memantine could significantly suppress the appetite and binge-eating disorder and finally decrease the body weight within a few days (5). In our results, compared to the saline group mice, the percentage of body weight in mice fed with standard control food diet and administered with 20 mg/kg memantine showed a significant decrease during the early memantine injection days but was similar in later injection days. Meanwhile, the percentage of body weight in mice administered with 5 mg/kg memantine injection was similar with that of saline group mice, while higher in some days. There are few reports about whether memantine affects the weight of people with healthy weight. Under standard institutionalized diet, Venturelli et al. found that BMI decreased significantly in Alzheimer's Disease (AD), while in CTRL it remained unchanged with similar levels of daily energy expenditure. The combination of three factors, number of medications taken, albuminemia, and cortisolism, predicted ΔBMI in Woman with AD (17). Several studies have reported NMDAR signaling in the regulation of appetite (18–22). NMDAR signaling regulates food intake at several appetite-suppressing nodes, including the solitary tract nucleus (23–25), the parabrachial nucleus (26, 27), the ventromedial nucleus of the hypothalamus and the paraventricular nucleus of the hypothalamus (22, 28), and the lateral habenula (29). In another study, the central amygdala (CeA) region was shown to play an important role in appetite regulation (13). Further research needs to be carried out to elucidate which brain areas are involved in the mechanism of memantine on obesity. Because of the important role of peptides (like leptin and ghrelin) in appetite related brain areas like hypothalamus, the expression of these peptides in brain may change.

Our results showed that memantine decreased the weight of obese mice by suppression of food intake. In the CTA model, memantine had similar effects to LiCl. Traverso et al. reported that MK-801, another NMDAR antagonist, induced low intensity conditioned taste aversion (30). MK-801 was reported to virtually block all NMDAR activity and manifested unacceptable side effects (31). Differently, memantine preferentially blocks excessive (pathological/ extrasynaptic) NMDAR activity and its activity remains mostly normal (physiological/synaptic) due to an uncompetitive mechanism of action in conjunction with a relatively fast off-rate, resulting in a low affinity for the NMDAR (31). Combined with our findings that memantine decreased food intake in CTA model, we suppose that the mechanism of memantine that suppresses food intake may be different from that of MK-801.

In the CTA model, our results showed that memantine had similar effect to LiCl. As we know, LiCl can induce abdominal discomfort. If memantine did induce abdominal discomfort, it would suppress the locomotor activity of mice in open field test. Interestingly, our results showed that LiCl group mice had less locomotor activity than saline group mice, while memantine didn't suppress but increased the locomotor activity in open field test. And memantine wasn't shown to cause anxiety. There are many studies about the effects of memantine on locomotion. When the time periods of open field test differ, the results can be different. In the 5 min open field test, the total distance traveled by rodents with and without memantine injection is similar (32). During the first 60 min open field test, Costa et al. found that the total distance increased following the increase of memantine doses (33). In the last 5 min of the 30 min open field test, Kotermanski et al. found that the total distance traveled by rats was increased following the increase of memantine doses (34). The duration of the period spent in the center of the open field could reflect the anxiety level in open field test, and the results among different studies differed. In the 5 min open field test, Camarasa et al. found that memantine-treated rats spent longer time in the center and shorter time in the periphery (35). When analyzed with different parameters or when the method of memantine intake differed, the anxiety level in the open field could differ (35, 36). Our results in plus maze showed that the percentage of duration in open arms (compared to duration in total arms) among different memantine groups was similar.

Foltin et al. reported that memantine decreased the food intake by enhancing the satiation (37). In our results, unlike LiCl suppressed locomotor activity, CCK group mice performed like saline group mice in open field test. We hypothesize that the decreased food intake and increased weight loss caused by memantine might be due to satiation.

Our results showed that memantine increased the locomotor activity in open field. It has been well known that exercise can improve health. In an elegantly designed study, Ross et al. (38) reported that the diet-induced and exercise-induced weight loss groups showed approximately 8% weight reduction, and had significant reductions in total fat mass, visceral fat and increased glucose disposal. However, when compared to the diet induced weight loss group, exercise training induced weight loss group had a greater reduction in total fat mass (39). In the sixth century B.C., Susruta advocated exercise as a treatment for diabetes (40). Muscle contractions and exercise increase energy consumption, glucose uptake (41, 42) and sensitivity of muscle to insulin (43). Adipose tissue and liver are also targeted by exercise. Adipose tissue is an active endocrine organ (44) that is dramatically influenced by exercise (45). Similarly, the liver helps mediating the beneficial effects of exercise (46). So, increased locomotor activity by memantine might lead to improvements in glucose homeostasis and decreased markers of liver damage in obese mice. Further studies are needed. However, Zimmer et al. found that long-term administration of memantine could induce anxiety-like behavior (47).

## Conclusion

Long term NMDAR antagonism by memantine increases weight loss in mice obesity induced by high fat diet. Memantine decreases food intake without inducing abdominal discomfort and anxiety, suggesting that this compound would be a good candidate drug for obesity control. However, the molecular mechanism and brain circuit involved in the regulation of weight loss by memantine need further study.

## Author Contributions

W-GL and FL designed the study and modified the manuscript. S-ND conducted the study and prepared the manuscript. Y-HY helped conduct the weight loss experiments and modify the manuscript. T-LZ and B-KM helped perform food intake experiments. H-RF and Y-ML helped finish open field tests and plus maze test.

### Conflict of Interest Statement

The authors declare that the research was conducted in the absence of any commercial or financial relationships that could be construed as a potential conflict of interest.
